# Comment on: "Correlation of pelvic ultrasonography with pubertal development in girls"

**DOI:** 10.61622/rbgo/2025rbgo72

**Published:** 2025-10-21

**Authors:** Amnuay Kleebayoon, Viroj Wiwanitkit

**Affiliations:** 1 Private Academic Consultant Samraong Cambodia Private Academic Consultant, Samraong, Cambodia.; 2 Saveetha University Saveetha Institute of Medical and Technical Sciences Department of Research Analytics Chennai India Department of Research Analytics, Saveetha Dental College and Hospitals, Saveetha Institute of Medical and Technical Sciences, Saveetha University, Chennai, India.

Dear Editor,

The publication on "Correlation of pelvic ultrasonography with pubertal development in girls"^(1)^ is interesting. The purpose of this study was to investigate the association between pelvic ultrasound pictures and pubertal development in girls, as well as to assess the efficacy of various factors in predicting puberty, particularly the use of uterine Doppler values, a relatively unknown indicator. Although the cross-sectional research approach is appropriate for investigating exploratory links throughout time, its primary disadvantage is the inability to determine causality. Furthermore, considering thelarche (the commencement of breast growth) as a single criterion for grouping may be insufficient because sexual development in females is complex and subject to individual variation.

Although a sample size of only 60 people is adequate for a preliminary ROC analysis, it is nevertheless regarded relatively small when considering age variety and quickly changing biological traits during childhood and adolescence. Furthermore, it is unclear whether confounding variables like body mass index, hormone levels, or other health issues that may alter ultrasonography values, such as ovarian or uterine volume, were controlled.

In terms of statistics, using AUC and the Youden Index to estimate each parameter's cut-off value is suitable and customary. However, the results of uterine Doppler analysis (PI ≤ 2.75) with a sensitivity of 100%, specificity of only 48%, and low PPV of only 62% suggest that this indicator may not be accurate enough as a single screening tool. It may also cause a large number of false positives in real practice, which must be carefully considered.

Further discussion questions include: To what extent do physiological differences in females of various ages influence uterine or ovarian volume values? Should their use in practice be based on age or body composition? Should Doppler be integrated into the overall examination, together with clinical or biochemical data, to improve the accuracy of appropriate puberty diagnosis? According to a fresh interpretation, the very high NPV (100%) of uterine PI may indicate that if the PI remains high, the child has not entered puberty, which is more appropriate for "exclusion" rather than "inclusion" in the first diagnosis.
