# Supplementary material for: Comment on: "Correlation of pelvic ultrasonography with pubertal development in girls"
Source: Rev Bras Ginecol Obstet. 2025 Oct 21;47:e-rbgo72. doi: 10.61622/rbgo/2025rbgo72 (PMC12671681; doi:10.61622/rbgo/2025rbgo72)
Supplement: Related texts

# Author´s reply to comment on: "Correlation of pelvic ultrasonography with pubertal development in girls"

**Authors:** Francine Zap Bertoncello, Mariane Faccin Beust, Cláudia Mendes Tagliari, Liliane Diefenthaeler Herter, Cristiane Kopacek

**Affiliations:** 1 Universidade Federal de Ciências da Saúde de Porto Alegre Porto Alegre RS Brazil Universidade Federal de Ciências da Saúde de Porto Alegre, Porto Alegre, RS, Brazil.; 2 Hospital da Criança Santo Antônio Pediatric Gynecology Unit Porto Alegre RS Brazil Pediatric Gynecology Unit, Hospital da Criança Santo Antônio, Porto Alegre, RS, Brazil.; 3 Hospital da Criança Santo Antônio Radiology Service Porto Alegre RS Brazil Radiology Service, Hospital da Criança Santo Antônio, Porto Alegre, RS, Brazil.; 4 Universidade Federal de Ciências da Saúde de Porto Alegre Departament of Gynecology Porto Alegre RS Brazil Departament of Gynecology, Universidade Federal de Ciências da Saúde de Porto Alegre, Porto Alegre, RS, Brazil.; 5 Irmandade Santa Casa de Misericórdia de Porto Alegre Endocrinology Service Porto Alegre RS Brazil Endocrinology Service, Irmandade Santa Casa de Misericórdia de Porto Alegre, Porto Alegre, RS, Brazil.; 6 Universidade Federal do Rio Grande do Sul Departament of Pediatrics Porto Alegre RS Brazil Departament of Pediatrics, Universidade Federal do Rio Grande do Sul, Porto Alegre, RS, Brazil.

Dear Editor,

We are deeply grateful for your interest in our work. Regarding the comments received, we would like to make the following remarks. Thelarche as a criterion for puberty is indeed not the best parameter to define the onset of puberty. We are aware that, in many cases, thelarche may be isolated, without activation of the hypothalamic-pituitary-ovarian axis, or may be secondary to the action of estrone in obese girls, or even be mistaken for adipose tissue on physical examination. The best parameter for comparison is always the gold standard, which in this case is the GnRH or GnRH analogue stimulation test.^(1)^ Unfortunately, in Brazil, this test is costly, and the financial resources for this research were limited. Moreover, many patients underwent the exam for reasons other than pubertal assessment, which would raise ethical concerns about using a medication—never free from adverse effects—in children without clinical indication.

Our sample of 60 participants was smaller than we had hoped. However, since this research was linked to a Master's program (with a duration of two years), there was a limited time frame for data collection. Another shortcoming was that we did not perform a sample size calculation prior to starting the study.

Based on the results of our study, we believe that pelvic ultrasonography may have an even more relevant role in the evaluation of precocious puberty than it currently does. We observed that simple ultrasound parameters, such as uterine and ovarian volume, can predict puberty with good accuracy. Indeed, the uterine artery pulsatility index (PI), when considered in isolation, is not a reliable parameter. Other studies evaluating PI have also shown that it does not outperform simpler and more routinely used ultrasound measurements.^(2-5)^ We also agree that the uterine artery PI may have a more relevant role in ruling out puberty due to its high negative predictive value.

We fully agree with the points raised, such as the influence of age and body composition, and these should indeed be taken into account in future studies. We are very pleased with the comments received, which will certainly contribute to improving the quality of our work.

## References

[1] Brito VN, Spinola-Castro AM, Kochi C, Kopacek C, Silva PC, Guerra G (2016). Central precocious puberty: revisiting the diagnosis and therapeutic management. Arch Endocrinol Metab.

[2] Laursen EM, Holm K, Brocks V, Jarden M, Müller J (1996). Doppler assessment of flow velocity in the uterine artery during pubertal maturation. Ultrasound Obstet Gynecol.

[3] Ziereisen F, Heinrichs C, Dufour D, Saerens M, Avni EF (2001). The role of Doppler evaluation of the uterine artery in girls around puberty. Pediatr Radiol.

[4] Paesano PL, Colantoni C, Mora S, di Lascio A, Ferrario M, Esposito A (2019). Validation of an accurate and noninvasive tool to exclude female precocious puberty: pelvic ultrasound with uterine artery pulsatility index. AJR Am J Roentgenol.

[5] Cheuiche AV, da Silveira LG, Escott GM, Lucena IR, Puñales M, Costenaro F (2022). Accuracy of Doppler assessment of the uterine arteries in healthy girls for the diagnosis of pubertal onset. Endocrine.
